# New Researches upon Arterial Murmurs, and the Application of Such Researches to the Study of Several Diseases

**Published:** 1846-10-01

**Authors:** 


### Nouvelles Recherches sur les Bruits des Arteres et Application de ces Recherçhes a l'Etude de plusieurs Maladies. Par le Dr. *J. H. S. Beau*, Medecin des Hôpitaux, &c. [Archives Generales, 1845-6.]

### New Researches upon Arterial Murmurs, and the Application of such Researches to the Study of several Diseases. By *J. H. S. Beau*, M.D.

Dr. Beau has just completed a series of interesting papers upon the subject of arterial murmurs, a succinct but complete analysis of which we propose to offer in the present article. They are, indeed, but a continuation, or rather extension, of a former essay upon the same subject published in the *Archives Generales* in 1838,* in which the two following propositions were maintained: *viz.* 1. Arterial murmurs depend upon the excessive friction which the blood exerts upon the arterial parietes, when this liquid exists in too considerable a quantity relatively to the capacity of these vessels. 2. Whenever, in any disease, arterial murmurs are heard which cannot be explained by a local lesion of the vessel, we may conclude that this diseased condition is accompanied by a superabundance of the mass of the blood, *i. e.* by a *polyæmia.* The present series of essays is devoted to a fuller development of these propositions.

### I. History of Arterial Murmurs.

This is divisible into a *descriptive* and *theoretical* portion, each of which occupies the author at some length, as fundamental to his examination of the various diseases in which the murmurs are heard. a. The *description* embraces that of the form, the seat, and the rhythm of the murmurs.

1. *The Forms of Arterial Murmurs.*—The varieties of these are nearly the same as those of the heart. Thus there is the short, instantaneous murmur, giving to the ear the sensation of a true impulse, which may be termed the *normal* arterial murmur: but this may be more or less prolonged, imparting an idea of friction, possessing the same shades of difference as the anormal sounds of the heart, and is then an *anormal* murmur. If, however, the *normal* sounds of the heart and arteries resemble each other in the sensations they impart to the ear, the perception of these latter is not very common. It is true we may usually hear normal sounds over the carotid and aorta, but, if we conduct our examination very accurately we shall find that these sounds are generally but the reflection *(retentissement)* of the first sound of the heart, heard sometimes over the aorta, and at others over the carotids. We can only be certain of the arterial sounds when we hear them beyond the reach of this reflection, as

* Vide Med. Chr. Review, Vol. xxix, p. 569.

*e. g.* over the femoral artery, or especially over the abdominal aorta, when the parietes of the abdomen are thin enough to admit of satisfactory auscultation. These vessels usually offer a pulsation or heaving, of a notable intensity, but this is very rarely accompanied by any sound. The normal sound is then exceptional, and even when it is well-marked it is usually connected with pathological conditions which furnish also anormal murmurs—so that in such arteries we may have simultaneously the one and the other.

The *anormal* murmurs present many shades which resemble the sounds whose names they have borrowed, as blowing-sound, sawing-sound, &c.; but these differences are by no means so important as they might seem, as they do not indicate corresponding varieties of pathological conditions, but may be dependent upon the different intensity of the same cause, and may be transmuted into each other according to the diminution or increase of such intensity, and often under the influence of ephemeral circumstances, as will be presently seen.

A marked *vibration* or *thrill* is usually conveyed to the finger placed over an artery that furnishes an anormal murmur, varying with the intensity of the sound. Whenever such vibration is felt we are sure of detecting a marked anormal murmur by means of the stethoscope.

2. *Seat of Arterial Murmurs.*—Arterial murmurs may be divided into *Local* and *General.* The former are manifested over a very limited portion of the arterial system, and result from some circumscribed affection, such as aneurism, aneurismal varix, erectile tumours, or the compression of arteries by tumours. The latter may be heard over many parts of the arterial system, and result from a general affection; and it is to these *General Anormal Murmurs* that we wish especially to direct attention. Although the general murmurs may usually be heard over several portions of the arterial system, this is not always the case; for oftentimes the murmur induced by even a general affection may be confined to one particular artery, which in such instances of limitation is invariably the same, viz. the *carotid.* We may add that the general murmurs frequently coincide with an anormal condition of the first sound of the heart.

3. *Rhythms of Arterial Murmurs.*—The arterial murmurs present varieties of these which it is important to recognize. 1. The murmur is only heard at each arterial dilatation (*the simple intermittent murmur*). 2. It may be heard twice during each revolution of the heart's action, the first sound being the stronger; between which and that which succeeds it there is a distinct pause (the *double intermittent*). 3. The murmur may be heard throughout the whole continuance of the revolution of the heart's action (*continuous murmur*). 4. It may not only be continuous, but at each manifestation of the pulse it may be redoubled or re-inforced (*continuous* and *redoubled murmur: bruit de diable* of Bouillaud). In general affections of the arterial system, in which several of the arteries are the seats of anormal murmurs, those which are nearest the heart usually affect one of the three last varietes of rhythm, while those which are more distant only manifest a simple intermission.

*Circumstances influencing the production or modification of the Murmurs.* —For the murmurs to be heard, the artery should be near the centre of the circulation. When they are heard in several vessels they are always louder in those of the neck and trunk than in those of the limbs, while, when they are confined to one vessel, that is never an artery of the periphery. For the murmurs to be heard the artery must be superficially placed, as the carotid, femoral, subclavian, brachial, or the aorta, in a retracted state of the parietes of the abdomen. When a deep-seated artery like the iliac furnishes a murmur, it is only through its being subjacent to a tumour, which conveys the sound like a stethoscope. The *Carotid,* being both superficial and near the heart, is that in which murmurs are most frequently heard. The stethoscope should be placed between the separations of the sterno-cleido-mastoideus, and the chin raised and turned to the opposite side, so as to impart a sufficient tension to the parts. The degree of *pressure* exerted upon the instrument should vary, being considerable when the sounds are obscure, and very slight when they are intense, for anything beyond simple *apposition* will then cause them to disappear. Whatever, too, operates a change in the more or less superficial position of the carotid will modify the murmur. Thus, it is heard with more intensity (and sometimes exclusively) on the *right side,* owing to the deeper-seated situation of the left carotid, except in those rare instances in which this is more superficially placed than the right. If we apply pressure against the artery above the point where the stethoscope is placed, so as to thrust the vessel into the deep angle at the side of the larynx, the murmur either ceases or becomes diminished in intensity according to the degree of pressure employed. The same results follow if we make due compression with the instrument itself. By another mode of proceeding, however, we may produce opposite effects. The carotid, and especially the left one, may lie deeply hidden in the angle at the base of the neck. If the index finger be passed in front of the anterior insertion of the sterno-mastoideus into the little fossa above the median line of the sternum, the vessel may frequently be raised by it into a more superficial situation under the mouth of the stethoscope, and sounds before unheard may be distinguished, and others of an obscure character rendered more distinct. The artery is, however, sometimes too deeply situated to admit of this manœuvre, and if it be employed upon a vessel which is superficially seated and already gives distinct sounds, the pressure it produces will be the means of diminishing or abolishing them. M. Donné has observed that traction of the larynx diminishes the carotid murmur, and attributes this to the point of support having been removed from the vessel; but the traction in fact withdraws the artery to without the sphere of action of the stethoscope, and the pulsations of the vessel may also be observed to be less and less perceptible in proportion to its amount. Donné has likewise remarked that, when the patient makes any sudden muscular exertion, the murmurs are less distinctly heard or suspended. This, in fact, arises from the distended condition of the jugular and other veins during such exertion by reason of their prominence, leaving the artery in a less superficial position than it before held in its angular space. If the effort made be much less, the diminution or alteration of rhythm is likewise proportionally less. In some subjects no difference in the murmurs occur in consequence of the distension of the jugulars during exertion being insufficient to displace the carotids. Hope has noticed that the arterial murmurs are much more full and intense during and at the end of inspiration than they are in expiration. The veins, in fact, are flattened in the one action and distended in the other. It is, too, on account of the different degree of repletion of the veins that the murmurs are heard so much more distinctly in the erect, or sitting, than in the recumbent posture; and M. Vernois has observed that, if the head hangs down upon the trunk, they disappear altogether. Many individual differences, regarded as singularities in the auscultation of arterial murmurs, depend simply upon the variations of organization, or of the anatomical relations.

Other circumstances may modify the murmurs without causing changes in the anatomical relations of the vessels. Bouillaud observes, after Laennec, that when an individual engages in active muscular exertion the the murmurs are increased in intensity, and often change their form from blowing to sawing sounds, &c. But it is also important to note the fact of such exertions likewise markedly *increasing the force of the pulsations,* not only of the carotids but of every artery. What proves the exactitude of the relationship is the fact that it holds good under reverse circumstances; for the more the pulse is diminished in volume the less intense are the arterial murmurs. If the large cupping-glass of M. Junod be applied to the lower extremity the diminution of pulsation and murmur will be found to be coincident with that of the volume of blood. Three patients, in whom arterial murmurs had been very audible, suffered from hæmorrhage, which much diminished the violence of the pulse, and, as long as this continued small, the murmurs were quite imperceptible in two cases and nearly so in the third. Two old cachectic women, whose carotids furnished intense murmurs, were greatly reduced by diarrhœa, which led at once to a rapid fall of the pulse and disappearance of the murmur. When a chlorotic patient falls into a state approaching to syncope, during which a very small pulse exists, the murmur infallibly diminishes or disappears. The cases noticed by Laennec and Bouillaud in which the murmurs become suspended or changed from time to time, may mostly be brought within one of the above categories. Thus, a chlorotic and hysterical woman may have a paroxysm of hysteria, during which her muscular efforts may suspend the murmurs by distending the jugulars. Sometimes, again, the action of the heart is defective from mental emotions, the pulse is consequently small and the murmur absent.

B. *The Theoretical Portion.*—As a matter of convenience we may divide the *anormal murmurs* into local and general. It is an arbitrary proceeding, inasmuch as they depend upon an identical condition; but the local affections of the arteries being more accessible and intelligible, the explanation of the anormal murmurs is more easily made by their agency. The local affections which give rise to murmurs are, *organic narrowing of the artery, its compression, aneurism, erectile tumours, and aneurismal varix.*

1. The mechanism of the production of the murmur in instances of *narrowing of the calibre* of the artery by thickening, concretions, &c. is simple. The fluid exerts an exaggerated degree of friction against the constricted portion, inducing the anormal sound and a thrilling, perceptible to the hand ; the mechanism being identical with that which induces the cardiac murmur in instances of narrowing of the orifices of that organ.

2. *Compression* of an artery will induce a murmur just in the same manner.

3. *Aneurismal Tumours* induce murmurs and thrilling in one of the above modes. Sometimes the flow of blood is impeded by the organised coagulum, and sometimes by the compression of the arterial tube by the tumour itself. Another obstacle has not been sufficiently noticed, *viz.* the blood which the aneurismal cavity habitually contains. The tumour is always full of blood, which in a healthy state of the tube should be propelled on towards the capillaries, and which presents an obstacle to any additional quantity of blood coming from the heart. There is then a sum-total of the fluid too great relatively to the capacity of the vessel, and an exaggerated friction against the walls, furnishing the murmur and vibration, results.

4. *Erectile Tumours* are usually the seat of marked anormal murmurs and vibrations. On dissection they are found to be pervaded by thin, dilated, flabby, arteries, containing coagula—they having become, as in aneurism, deprived of their middle coat and contractile power. The distension of each vessel is at its maximum as often as a new supply of blood arrives within them, and the conditions for the formation of murmurs are then analogous to those prevailing in aneurism.

5. *Aneurismal Varix.*—The murmur and vibration proceeding from this exists not only over the opening of communication between the vein and artery, but is prolonged along the whole length of the former. The vein by its organization is unable to re-act upon the arterial blood which enters it, and the murmur and vibrations result from its consequent distension. If these are less along the continuity of the vein than over the aperture, it is because the arterial wave which penetrated the aperture with violence, so to say, exhausts itself against the obstacle which the venous blood offers to its progress.

From the above observations we may deduce this general principle, that the *primary condition of the production of arterial murmurs is a defective proportion between the wave of blood and the calibre of the artery.*

MM. Andral and Gavarret endeavour to explain the existence of these murmurs by their dependence upon a *defective proportion of globules in the blood.* Thus, if the globules are but 80 in the 1000 parts, the murmurs are constantly heard; from 80 to 100 they are heard frequently ; but they are never observed, in connection with changes of the blood, when the proportion exceeds the normal one of 127. There is doubtless some general coincidence between the murmurs and proportions of globules ; but do the murmurs depend upon this condition of the blood itself, or upon a disposition of the arterial system which usually accompanies it? We believe the latter. In fact, if the murmurs depended essentially upon the diminution of globules, they should always co-exist with blood so impoverished, and then we ought to be able to diminish the volume of the pulse in a chlorotic subject by means of Junod's cupping-instrument without diminishing or abolishing them, as by it no change in the composition of the blood could be effected. Numerous experiments, however, have proved that, by diminishing the volume of the pulse, we proportionally diminish the murmurs. On the other hand, MM. Becquerel and Rodier relate the cases of two girls who presented the *bruit de diable* in the carotids, the blood being of its normal composition. Similar observations will apply to the general rule laid down by M. Bouillaud, that arterial murmurs are found only in individuals *the density of whose blood is below* 6° of Beaume's areometer. The arterial murmurs, in fact, depend upon a condition of the arterial system which is found when the density of the blood is thus diminished.

Experiments, consisting of the injection of water into the veins also produce murmurs, and that in proportion to the degree of distension caused and the force employed. If the local affections of arteries and experiments agree in furnishing the same formula, viz. *a defective proportion between the wave of blood and the capacity of the vessel*, can it also be deduced in the case of murmurs connected with a *general affection of the arteries?* When we find the murmurs are much augmented by whatever distends the larger arteries and suppressed by diversion of the blood to the periphery, we may believe that in the mean of these two extremes there is a state of superabundance of this fluid, which constitutes the disease of which the arterial murmurs are a symptom. To illustrate this point M. Beau cites the phenomena attendant upon *insufficiency of the aortic valves*, and upon *chlorosis*; but as these are detailed in the second part of the Memoir we may defer noticing them. But before entering upon these we must advert to a long digression upon the

*Alleged Jugular Murmur.*—During forcible expiratory efforts the blood is driven from the central into the peripheral veins. In many persons, otherwise healthy, this reflux is attended with the production of anormal sounds and thrilling in the femoral vein or internal jugular. They are better heard when a sudden muscular effort is made rather than noisy expiratory acts, as coughing, &c. which may obscure them. These venous sounds, like the arterial ones, depend upon the disposition which exists between the volume of the blood and the calibre of the vessel. M. Beau adduces various arguments in refutation of the opinion entertained by Drs. Hope and Ward, and adopted by M. Aran, that the continuous murmurs heard in the carotid region have their seat in the *veins* of the neck. He maintains that the carotid is their true seat, and that they become more or less audible accordingly as the flattened or distended state of the jugular vein allows this artery to become more or less superficially placed. In fact, the only true *venous murmurs* that are produced result from aneurismal varix, or are temporarily induced during acts of expiration in the femoral or jugular veins.

### II. The various Diseases which are characterised by the Existence of Arterial Murmurs.

It is intended to shew, by the analytical examination of symptoms, or by experiments, that the arterial murmurs in these affections depend upon the presence of a superabundant quantity of blood; and to inquire into the pathological cause of such superabundance. The various affections are considered without any attempt at methodical arrangement.

### 1. *Insufficiency of the Valves of the Aorta.*

Insufficiency of the aortic valves forms a natural link between the local and general affections; for, although the lesion is local in its nature, it leads to general derangement of the arterial system. As a consequence of its existence, there is a reflux of blood into the left ventricle during the pause in the heart's action. This, together with the blood coming from the auricle, constitutes a superabundance of the fluid during the next action of the ventricle. This cavity becomes dilated and hypertrophied, and the blood is propelled with an exaggerated friction into the aorta. In this disease the pulse is full on account of this superabundance, and hard in consequence of the hypertrophy. Sometimes the thrilling which is perceived over the carotids is extended even to the radial artery, and induces a vibratory pulse. In insufficiency there is (1), an anormal murmur heard during the second sound of the heart, opposite the aortic orifice, and produced at the point of insufficiency by the reflux of blood into the ventricle by reason of the elastic re-action of the aorta. There is, (2), also an anormal murmur heard in the large arterial trunks, synchronous with the first sound of the heart, and generally accompanied by thrilling and considerable arterial pulsation. We are here speaking of simple insufficiency; for, if this become complicated with *contraction of the aortic orifice*, the wave of blood is impeded, and this last sound is absent. The anormal murmur arising from the reflux of the blood however remains, and is therefore the characteristic symptom of insufficiency. Indeed, we may say that, whenever the heart offers an abnormal murmur in its second sound, this certainly arises (except in pericarditis) from insufficiency of the aortic valves.

"There is an interchange of cause and effect worth observing. The elastic reaction of the aorta is the determining cause of the dilatation and hypertrophy of the ventricle; but in proportion as these become more considerable, so is the quantity of blood which re-flows into the heart larger. The wave expelled into the arteries becomes also larger and larger, and the arterial pulsations more marked. Such amplitude of pulsation cannot exist without dilatation of the calibre of the principal arteries. Thus the hypertrophic dilatation of the heart, which is at first produced by the arterial reaction itself, becomes a cause of arterial dilatation. The mass of the blood also does not continue the same. It is in excess: but as the portion in excess only executes a to-and-fro movement between the heart and arteries reciprocally, it follows that it is placed as it were beyond the pale of the circulation."

### 2. *Anæmia following Loss of Blood.*

The state of anæmia may seem to present difficulties for the explanation of the arterial murmurs by a condition of *polyæmia*.

"Let us first trace the principal marks of anæmia resulting from loss of blood. It is characterized by paleness of face, colourless tissues, smallness of pulse, and arterial murmurs. It depends alike upon the blood containing a large proportion of serum, and the entire mass of this fluid being less than in its normal state. This definition, which seems to be correct, is nevertheless inexact; for, in fact, it confounds under one name, as the same morbid entity, two conditions which should be essentially distinguished from each other. I will indicate these conditions as an anticipatory recapitulation of the facts which will presently come under our notice.

"1. A notable abstraction of blood at once produces paleness of face, small pulse, &c. There are however *no anormal arterial murmurs.* The symptoms are produced entirely by the entire mass of the blood being below its normal quantity—a condition of *true anæmia.* 2. After a certain time, varying according to the abundance of the loss of blood (one or two days, &c.), the pulse acquires a greater volume, and becomes much fuller than prior to the loss of blood, and from this period anormal murmurs may be heard. The fulness of pulse and arterial murmurs are both dependent upon a superabundant reproduction of serum. The entire mass of the blood has become more considerable. The condition is that of *polyæmia,* but of a *serous polyæmia.*"

If the loss of blood be very excessive, so as to throw the person into a state of excessive prostration, the *thirst* so usual after hæmorrhage will not be present, *drinks* are not taken, and the *excess of serum* not produced. The pulse will then continue small, and the patient sink without any arterial murmurs being heard. There is only one case in which the murmurs are heard immediately after the loss of blood, and that is when they were already audible before it took place—and not then if the hæmorrhage be very excessive. There are other symptoms indicative of polyæmia after hæmorrhage besides fulness of pulse, such as pulsative cephalgia, vertigo, dyspnœa, palpitations, &c.—all of which are increased by exercise and temporarily diminished by abstraction of blood. With such symptoms we have pallor and debility, because, although the blood is superabundant in quantity, it contains much too large a proportion of serum. Dupuytren has noticed the state of plethora with pallor and debility following the frequent loss of blood in hæmorrhoids.

To decide the nature of the *anatomico-pathological condition upon which the state of serous polyæmia depends,* the author instituted two series of experiments. The first of these, consisting in bleeding dogs from day to day, was only confirmatory of the observations already made, by the induction of murmurs after the fifth or sixth bleeding, and their disappearance after additional bleeding until the animals had the opportunity of recruiting the supply of serum by drink. The other series was performed on rabbits, and an endeavour made to compare the condition of animals who had been subjected to repeated depletion with others in their normal condition. An examination of the heart in these animals disclosed important differences, explanatory of the cause of the large wave projected into the arteries of those who had been frequently bled. In animals, previously strong and vigorous, and killed at once by opening the carotids, the cavities of the heart, and especially the ventricles, were found nearly effaced by the complete contraction of the organ. But in rabbits killed in the same way, but previously enfeebled by repeated bleedings, the organ was found of one-third more than its proper size, its cavities manifestly dilated and often containing blood. With the dilatation there was also hypertrophy, for, notwithstanding the dilatation of the cavities, their parietes were as thick as in the normal condition, while the weight of the organ was augmented by one-fifth or one-sixth. The origins of the pulmonary artery and the aorta were likewise notably dilated. We thus see that dilatation with hypertrophy (in practice it is familiarly known that hypertrophy may be produced when contraction of the orifices exist, notwithstanding the blood is defective in globules) may be rapidly developed under the influence of a poor, serous, fluid. This condition of the blood leads to an atonic and relaxed condition of the structure of the heart. A larger quantity of fluid being received into the dilated cavities, a condition of hypertrophy is essential for its propulsion.

Several passages are extracted from the writings of *Van Swieten, Lieutaud*, and *Lancisi*, shewing that the production of plethora after free bleeding was not unknown to them. The author likewise observes that, if we admit the reality of serous polyæmia, we are led to *question the efficiency of Valsalva's mode of treating aneurism*, viz. the institution of frequent bleedings and low diet, in order to enfeeble the patient's strength, and that of the wave of blood entering the sac. Such treatment will effect the former of these objects, but will only increase the intensity of the arterial pulsation. To ensure success by this mode, we should have to debar the patient from drinks, but thirst would be insupportable, and the consequent debility be incompatible with the maintenance of life. But when we have reason to *fear* the *recurrence* of *bleeding from wounds of arteries, operations for aneurism, &c.*, the prevention of an undue development of the pulse by a temporary abstinence from drinks seems to be a rational practice.

There are *two symptoms common to true anæmia*, and to *serous polyæmia*, viz. *paleness* and *debility*. The first of these, in both cases, is derived from a diminution of globules. In *true anæmia* this coincides with a proportionate diminution of the other elements of the blood; but in *serous polyæmia* it is accompanied with a disproportionate increase of serum. In the latter, too, there is a puffiness with the pallor of the face, while in the former the features are sunken and contracted. In *polyæmia* the *debility* is much less considerable than in *anæmia*, shewing that, although the reproduction of the blood is chiefly serous, such fluid will minister to some of the functions of the economy. *Syncope*, which is so characteristic of *anæmia*, is also sometimes observed in *polyæmia*, when, after muscular exertion, the blood arrives at the heart in too large quantities and surcharges it. It is then accompanied by palpitations and a sense of great weight, circumstances which do not prevail in anæmia. In both cases it arises from the heart being temporarily too feeble to propel its contents. The *duration* of these two conditions is very different. *Anæmia* is not of long continuance. At the end of a few hours the material taken as drink finds its way into the blood-vessels. We might almost say "*Nature has a horror of anæmia.*" *Serous polyæmia* may be of a much longer duration, but we may say, as a general rule, that in about a fortnight the normal proportion of globules will have become restored under the influence of aliments. On other occasions, however, it may be prolonged for months or even years, the digestive functions having undergone important and permanent alterations, and the consequent defective assimilation preventing the reparation of the impoverished condition of the blood.

### 3. *Chlorosis.*

The author presents a general view of the symptoms attendant upon this disease: but we will only extract those of them bearing reference to the condition of the circulation.

"There is a sense of weight at the heart accompanied by frequent palpitations: the organ is voluminous as I have assured myself by plessimetry. Its pulsations are increased in force, and frequently furnish to the ear the metallic tone, especially during the existence of palpitation. The normal sounds are clear and sufficiently intense to be heard over the entire chest. There is often a blowing sound accompanying the first sound of the heart. There are anormal murmurs in the arteries which are accompanied by thrilling, the pulsations, and especially of the carotids, being energetic and visible at a distance. The radial pulse, usually described as small and feeble, presents on the contrary *a more considerable volume than in the state of health,* the veins also being now distended, but of a less deep colour than normal. It has been said that the veins at the back of the hand are small in chlorotic persons, but they are still smaller in such persons in a state of health; and we must bear in mind that all the vessels are of a smaller calibre in a girl than in a man; and indeed the sole means of judging of their normal plenitude is to compare them during and after the cessation of the disease. * * * * * * * The proportion of globules of the blood in this disease is diminished from 127 to 38, while that of the serum has increased from 790 to 868. Little attention has been paid to the lesions of the solids, but it is admitted that the tissues are pale, slightly imbibed with serosity, and relaxed. It is said also that the heart is small and atrophied, but here preconceived ideas rather than observation have prevailed, for it is impossible that a heart shall be found small at an autopsy which percussion has proved to be developed.

"The full pulse, arterial pulsations, pulsatory headache, &c. of chlorosis depend upon the superabundance of the mass of blood, for (1). They diminish immediately, and for a while, if bleeding or Junod's cupping-glass be employed. (2). They are increased by exertion which throws a larger quantity of blood upon the centre of circulation. The heart re-acts upon this and drives the blood with additional force into the arteries, producing the sensations spoken of. At last it is unable to continue such exertion and the violent palpitations are then replaced by syncope."

Various authors, as Sennertus, F. Hoffman, Boerhave, &c. have noticed the superabundance of serous blood in chlorosis. The small proportion of globules is not only the cause of the colourless state of the tissues, but also of the languor and debility of chlorosis. It induces a relaxation of the principal tissues, as the muscles, skin, arteries, &c., and leads to the hypertrophic dilatation of the heart. In serous polyæmia the defect of globules arises from prior abstraction of blood, but in chlorosis it is due to defective assimilation. The disorder of the digestive organs, therefore, plays an important part in determining the condition of chlorosis, inasmuch as it is the point of departure of the changes which the blood undergoes: but in a case of simple chlorosis such disorder may itself be produced by prior derangement of the functions of the uterus.

"It is needless to observe that the various symptoms thus classed in their pathogenic order, are more or less masked in different individuals, and they may not even be constantly met with. I may add that they re-act more or less upon each other, so as to pass from the condition of effect to that of cause. Thus the affection of the digestive canal, which is the cause of the changes in the blood, may itself become increased by the diminution of the globules. The different alterations, functional or organic, too, whose succession and dependence I have shewn, may so combine that the effects are almost coincident with causes, so as to constitute an assemblage of phenomena which are exhibited and increased simultaneously.

"If we cast a retrospective glance at the symptoms of chlorosis and compare them with those of post-hæmorrhagic polyæmia, we find that the organico-pathological condition is identical in both. There is the same lesion of heart and arteries, the same change in the blood, and the same symptoms dependent upon the pathological condition of the blood and its reservoirs. In chlorosis the symptoms originate in an affection of the digestive and uterine organs; in polyæmia there is nothing of the kind, unless it becomes indefinitely prolonged. If the subject be a menstruating female, an uterine affection may be observed; but instead of this being the point of departure, as in true chlorosis, it is only one of the consecutive effects. As to nervous symptoms, such as neuralgiæ, they are seldom observed in post-hæmorrhagic polyæmia, unless this is very long in being dissipated, in which case they may become as intense and as numerous as in true chlorosis."

4. *The Cachexiæ.*

M. Beau enters upon the description of various forms of cachexia as the *Miners*, the *African*, the *Saturnine, &c.*—exhibiting the fact that all these depend upon the presence of a blood too poor in globules and too great in quantity—this, however, resulting from antecedent affection of the digestive organs impeding the assimilatory powers of the economy. The symptoms too are precisely analogous to those attendant upon chlorosis and post-hæmorrhagic polyæmia. He next offers several interesting *General Observations upon the Cachexiæ.* The term cachexia, or bad habit of body, was much employed by the older medical writers, during the reign of the humoral pathology, to indicate the condition of the system denoted by, among other symptoms, paleness of face, exhaustion of strength, and relaxation of tissue; but, even before the school of Pinel in its ultra-solidism had entirely rejected it, Cullen and Sauvages had completely perverted its original meaning, by applying it to a variety of dissimilar diseases in their classifications. Bordeu employs it almost as an equivalent to diathesis.

"At present the term is employed pretty frequently. This recurrence to an old name has taken place since the study of the pathological condition of fluids in disease has been resumed. Nevertheless its signification is not clear and precise, for by cachexia is often understood either a diathesis or some grave change in the organism consecutive to morbid causes which have long been inherent in the constitution of the individual. It is in this double sense we must interpret the scrofulous, syphilitic, tubercular, &c. cachexiæ so often mentioned in medical language. I wish to show that, if we desire to treat of the same affections as were heretofore indicated by this term, we must comprehend under it the same disease which is now called *anæmia* (Andral), *hydræmia* (Bouillaud), or *hydröemia* (Piorry)."

Several quotations are furnished from the writings of Cœlius Aurelianus, Paulus Ægineta, and other of the older authors, corroborative of the above views of the causes and symptoms of cachexia. From the period at which Lieutaud wrote (1769), however, we do not find the affection mentioned in treatises of medicine; but in our own times we may add to the above names in the history of cachexiæ those of Andral, Bouillaud,

Gavarret, and Piorry, who, under the names chlorosis, hydræmia, anæmia, &c. have described the same essential condition.

"To fix our attention upon the value and employment of these different terms, we think the word *anæmia* should be entirely restricted to designate the ephemeral state in which the quantity of the mass of the blood is considerably less than in the normal condition, as it is immediately after a great loss of blood. The term *hydræmia* should signify that alteration of the blood which consists in the diminution of globules and the acquisition of serum, with the preservation of the normal amount of fibrine. Lastly, under the term *cachexia* we may comprehend the disease itself, in which hydræmia shews itself with its characteristic symptoms. *Cachexia* is thus applied to the union of the alteration of the blood and the symptoms, while *hydræmia* designates the change of the blood alone. Every cachexia must necessarily arise from a hydræmia, but every hydræmia does not constitute a cachexia. We may define a cachexia to be an apyretic and usually chronic disease, characterized by the existence of a hydræmia which induces diminution of strength, and colourlessness and atony of the tissues."

Among the various species of cachexia thus defined we may enumerate the *syphilitic* (Ricord and Sennertus), the *paludian* (Hoffman and Bretonneau), that of *prisoners* (Sennertus), and that arising from *moral causes.* Certain *localities* will give rise to it, and thus Sir J. Clark describes a form of cachexia resulting from prolonged residence in London, and the same may be done in respect to Paris. A *verminous* cachexy is often observed in children and sometimes in adults. It may be induced by *bad food,* and become epidemic in time of famine. The workers of *lead* are liable to the saturnine form; and *cooks* are another class of persons largely subject to it. Certain *articles of diet* will produce it, and numbers die in a chlorotic state at Lille, and other parts of the north of France, from drinking corn or potatoe brandy. Smoking *opium* and the too long use of *mercury* lead to the same results. Cachexia may depend upon an *anterior pathological condition,* as cancer, arising not from this impregnating the system, but from the hydræmia it generates when it gives rise to hæmorrhage or enfeebles the digestive organs. Other cachexiæ result from excessive fatigue, seminal emissions, undue lactation, and other exhausting causes. The term *chlorosis* is applied too indiscriminately, whether diseases of the genital or other functions be the original cause. M. B. suggests its abandonment and its replacement by that of *virginal cachexia,* adding the name of its presumed cause. Thus it might be induced by dysmenorrhœa, asthænia of the generative organs, imperfect assimilation, moral causes, &c. In this way, too, we might dispose of the question of the existence of chlorosis in men.

Having attempted to render the application of the term more precise, the author next offers various general observations. We have already seen that all persistent cachexiæ become eventually dependent upon a disordered state of the digestive organs as their material centre—such disorder being either latent or evident, and arising from direct or indirect causes.

"It will be easily understood that the degree of atony necessary for the production of cachexia will not show itself in all individuals in the same degree from a like loss of blood-globules. Individual susceptibilities vary much in this respect. Thus, man, with his strong-toned tissues and powers of nervous resistance, becomes cachectic in a smaller degree than woman. We see, too, why arterial murmurs do not always correspond in intensity with the diminution in the amount of globules. It is only when the globules are reduced below 80 per 1000 that murmurs are invariably heard, because then the blood is so impoverished and unstimulating that, let the individual possess what tonic reactive power he may, the tonicity of the heart and arteries will still yield: although above this figure they may do so in very various degrees in different individuals. So, too, M. Bouillaud has found that the *bruit de diable* may be always heard in individuals, the density of whose blood is diminished to below 6° of Beaume's areometer. * * * * * * * *

"It is commonly insisted upon that a great resemblance exists between cachectic affections and diseases of the heart, and we are recommended not to confound them together. This is not surprising, seeing that there is really a material lesion of this organ in cachexia; but it does not furnish the same therapeutical indications, for we know that it is consecutive to the changes in the blood, and that it disappears when the normal composition of this fluid is restored.

"*Iron* is an agent almost exclusively employed in the treatment of cachexia, and its use has been in part recommended on the ground of the deficiency of its normal proportion in the blood. It is believed that the administration of ferruginous preparations may restore the normal quantity of the metal to this fluid, and on this ground the most soluble preparations are recommended, and, according to M. Mialhe, those chiefly should be chosen which are susceptible of decomposition by the alkalis of the blood."

M. Beau doubts the correctness of this view of the direct agency of iron, although the benefit of the substance is incontestible. The iron of the blood is contained only in its globules, and there will be more or less of it as these globules prevail. To cure hydræmia, therefore, and augment the proportion of globules, it can never suffice to introduce one element of the globule only. The diminished proportion of globules is maintained by the defective condition of the digestive functions, and iron acts only by restoring these to their integrity. In fact, we daily witness cases of chlorosis treated by iron in its most soluble forms without any success whatever—the digestive organs in such cases being in a condition not admitting of their benefiting by its agency. The iron is abundantly absorbed into the blood, and yet the globule is not constituted. On the other hand, when the metal acts beneficially, the earliest effect it produces is upon the digestive organs. We see other patients, again, recover under the use of aloes, change of air, or the removal of moral causes, &c., without ever taking iron at all, or after abandoning it as useless—the dyspepsia having yielded to other means after the iron had failed in relieving it. Our primary indication then must be to attack *the cause which produces the dyspepsia*. M. Trousseau has already protested against this indiscriminate treatment of the cachexiæ by iron; and, in fact, we can only re-constitute the blood by re-establishing the digestive functions, and removing causes which operate injuriously upon them. It is not by the direct agency of this medicine, but by the greater amount of aliment it enables the digestive organs to master, that iron is useful; and cachexia induced by mere hæmorrhage may be at once removed by the rapid administration of aliment alone.

The observations already made will sufficiently show that the fulness of pulse and excess of blood in these cases are not to be met with bleeding, although this produces a temporary alleviation. But in exceptive cases, of paralysis from congestion of or hæmorrhage into the nervous centres, it may be required, also when dyspnœa and palpitation become so urgent as to menace life. In these rare cases such congestion must be diminished either by the abstraction of blood or by the employment of Junod's cupping instrument. "This precious instrument, which acts more powerfully than the abstraction of blood, without causing that loss of the fluid always to be so much regretted in cachexia."

5. *The Menstrual Epoch—Amenorrhœa—Pregnancy.*

"The menstrual flux does not appear to be derived from the general mass of the blood, which in normal menstruation would then be proportionably diminished, but from an excess of blood which is produced during the interval of menstruation. This is proved, (1), by the weight and painful sensation in the loins, groins, and hips, which the woman experiences just prior to the epoch. (2). This uneasiness, resulting from an uterine congestion, begins to dissipate at the same time that the flux appears. (3). It does not exist at the end of this epoch, when the woman feels better than ever. (4). When the menses do not appear it increases more and more, and generally persists until they are established.

"If the phenomena of menstruation are confined to the symptoms of uterine congestion just mentioned, there will be found no arterial murmurs, and this is most commonly the case. But in other cases to these are added other phenomena, which are manifested in the different systems, as cephalalgia, vertigo, dyspnœa, palpitations, swelling and heat of face, &c., the pulse is full, the pulsatory movements of the arteries very visible, the veins distended—most of which symptoms are aggravated by exercise. In these cases the murmurs are heard in the carotids, appearing or disappearing with the preceding symptoms. They exist three or four days prior to the sanguine flux, and are dissipated before the termination of the menstrual epoch."

The existence of the connexion between polyæmia and the anormal arterial sound during menstruation is also shewn by the fact of their being exactly proportionate to the fulness of the pulse, becoming less when it is small, or even imperceptible, except during the production of some temporary physical or moral excitement.

*Amenorrhœa.*—In those instances in which there are general symptoms there are arterial murmurs dependent upon the presence of an augmented quantity of blood, but without the serum being in disproportion, as in chlorosis.

"Other forms of amenorrhœa are observed, in which, notwithstanding that plethora is evident, the arterial murmurs are yet not heard. In fact, for the production of murmurs, polyæmia is necessary, but every polyæmia will not induce them. In order that anormal sounds may be produced by the excess of blood, there must be hypertrophic dilatation of the heart, when the blood sent forth by it cannot traverse the vessels without exciting an exaggerated friction against their walls. But if the excess of blood only exist in the capillaries of the lungs, brain, or other organ, there is no reason why the murmurs should be induced in the large arteries; and in this case we do not see the arterial pulsations which are observed to be so violent after exercise in persons affected by arterial murmurs. There may then be amenorrhœa with a dilatation of the heart, and an excessive wave of blood characterised by the arterial murmurs. In other cases, again, the plethora is only manifested by local congestions of one or several organs, and as the wave sent forth by the heart is not more voluminous than normal, it traverses the arteries without exciting the vibrations and accompanying murmurs."

*Pregnancy.*—The polyæmia of pregnancy is a serous one, for Andral and Gavarret have shewn that the blood during this condition contains more water and less globules than are normal—the blood, however, being far less serous than in cachexia, properly so called. This excess of blood explains the occasional occurrence of cerebral hæmorrhage prior to, or during, labour. They may also occur subsequently to it, if the uterine hæmorrhage which follows childbirth generates a puerperal cachexia. M. Larcher observed, in 1828, that in pregnancy there was a notable degree of hypertrophy of the heart. This condition need not surprise us, seeing that the existence of anormal arterial murmurs presupposes dilatation of the heart with more or less hypertrophy. Still, at the author's request, M. Ducrest made additional investigations upon the subject in 1843, and furnished him with a statistical table of the results.

" This table is composed of the particulars of 100 women, for the most part between the ages of 20 and 30, who died during child-birth. In all of them the measurements of the heart were taken at the thickest part of the left ventricle. The maximum was 0018 millimeters in five cases, rising in one to 0022m. The lowest figure was 0011m. in eight cases: in most it was 0016m; the mean of the whole admeasurements being 0015m. If we compare these with that assigned by Bizot (0010m.) as the normal mean of this portion of the heart in the female, we have an excess of 0005m."

M. Beau believes M. Bouillaud's explanation of the cause of the murmur in pregnancy, viz. *the compression of the iliacs by the distended uterus*, to be the correct one.

6. *Fevers.*

Certain febrile affections present arterial murmurs, which are explicable upon the same pathological principles already stated. The murmurs in *Typhoid Fever* have been attributed to its accidental complication with chlorosis, or to the loss of blood during the treatment of the disease. The author, on the contrary, believes that these arterial murmurs are an essential feature of the disease, and he examined this point carefully in all the cases he met with last year. Having separated all the women who might have been chlorotic, and such of the men as had undergone bleeding, he found of the 25 recent cases which remained, the murmurs were heard in all but four. In these patients there was also a fulness of the pulse and enlargement of the heart—the state of this organ being compared in some cases during the fever and after its cessation. In those forms of the disease in which copious evacuations were induced, the pulse, volume of the heart, and arterial murmur, simultaneously diminished. The symptoms and the cadaveric lesions alike testify to the existence of a state of polyæmia. The heart and large arteries are found dilated, and various organs much injected. The best observers differ as to the change in its composition which the blood has undergone in this disease. *Typhus Fever* and *Yellow Fever* offer like evidence of the same condition of full pulse and voluminous heart, whence the author infers they must furnish the murmur. Since he has turned his attention to this subject he has met with seven cases of

*variola* in men, in all of whom the murmurs were heard. They were intense during the preliminary and the suppurative fever, but much less so during the interval occupied by the coming out of the eruption. The polyæmia which exists in variola does not exist in *scarlatina simplex* or *rubeola*, in neither of which are the murmurs heard. In *ague* the murmurs are to be heard, especially during the hot stage.

7. *Scorbutus and Purpura.*

Four cases of *Scorbutus* observed by the author plainly furnished the murmurs, and these explain the existence of certain symptoms, such as dyspnœa, palpitation, cephalgia, vertigo (all increased upon the slightest exertion) which, together with hæmorrhages, have been considered as characteristic of this disease. In these cases, too, there were fulness of pulse and arterial vibrations, so that a state of polyæmia existed. Andral has shewn that the blood is deficient in fibrine, which accounts for its insufficiently exciting the various tissues, and the consequent atony and dilatation of the heart. *Purpura,* with the exception of the affection of the gums, is analogous in all respects to scorbutus : and when it is generalized we observe the full pulse, arterial murmurs, vertigo and the like. Although hæmorrhage in typhus, scorbutus, &c. may in some degree depend upon the defibrination of the blood, they also depend upon its superabundance.

8. *Dropsies.*

It is intended here only to allude to those forms of dropsy termed essential, and which depend upon an altered condition of the blood. 1. *Dropsies with Albuminuria.* In these the arterial murmurs are habitual. Of 12 cases, occurring in men who had not been long under treatment, the author observed them in 10, the other signs of polyæmia being also the same as those seen in typhoid fever and cachexia. Nearly all observers have indeed noted in these cases a dilatation of the heart with or without increase of its substance. In 100 autopsies Bright only found the organ in its normal state in 33. The polyæmia existing in this disease has been directly adverted to by Martin Solon alone, who admits a serous plethora in cases wherein the pulse presents fulness. 2. *Dropsies without Albuminuria.* These are much more uncommon than the others. The author has met with four, however, in which the murmurs and other symptoms of polyæmia existed. The symptoms present often lead to this dropsy being considered as arising from disease of the heart, while in fact this organ is not more frequently diseased in these cases than in Bright's disease. Dropsies are, however, here alluded to only which are not complicated by venous obstruction or asthma, and in which the heart is not diseased at its orifices—a simple hypertrophic dilatation of its walls only existing. The author believes that the derivation of albumen to the kidney or its insufficient secretion may give rise to these two forms of dropsical effusion ; but he also attributes much to the mere mechanical extravasation which results from the state of polyæmia. At the commencement of the disease a free bleeding suffices to dispel the œdema, which returns as soon as the state of polyæmia is reproduced.

9. *Hypochondriasis.*

Laennec first observed the arterial murmurs in this disease, and it is singular that it should be the only affection in which he proved its existence, although there are so many others in which it is of far more common occurrence. M. Beau cites at considerable length the description given by Highmore, and has been enabled to confirm its accuracy both in private and hospital practice, it being met with in the latter more frequently than is believed, but some one of its predominant symptoms is confounded with the names of other diseases, as gastralgia, flatulent dyspepsia, anæmia, hypertrophy of the heart, chronic gastritis, cerebral congestion, &c. An increased fulness of pulse, enlarged volume of the heart, and arterial murmurs have been observed in all the cases furnishing symptoms as detailed by Highmore. The blood, too, is *serous*, having lost its globules during the mal-assimilation by the digestive organs. The *hydræmia* is, however, much less considerable than in true cachexiæ, and should perhaps be rather regarded as the first stage of this, the blood possessing an analogous composition to that of a pregnant female. The blood possessing a less tonic property than normal, the same series of changes in the heart, so repeatedly referred to, take place; the nervous debility of these persons assisting this impoverished blood in inducing atony of the organ. M. Beau agrees to the pathogeny of Highmore, who places the original seat of the disease in the enfeebled digestive powers, which cease to elaborate properly; a serous blood resulting. Highmore also shews that there is an excess of such blood in the system, and attributes to the distension of the vessels, which he supposes this to cause, all the subsequent symptoms of the disease.

*Hypochondriasis* much resembles *Cachexia* in its nature, chiefly differing from it in the lesser amount of impoverishment of the blood. Hypochondriasis has been said to be especially met with in man, but in women the same form of symptoms is often confounded under the terms amenorrhœa, dysmenorrhœa, and especially chlorosis. Indeed, the greatest difficulty sometimes exists in distinguishing between the two affections, and far more than exists in distinguishing between hypochondriasis and hysteria, which has occupied so much attention.

Among the cortege of symptoms enumerated by Highmore and others, the fears, dread, and delusions of hypochondriacs respecting their health and the gravity of the diseases they may be the subjects of, always figure; and, by the more modern writers upon this disease, these have been especially seized upon as its essential and almost sole characteristic.

"Dating from this epoch (the end of the 18th century) hypochondriasis has become, so to say, entirely absorbed and effaced by one of its habitual symptoms—the peculiar perversion of the understanding, which consists in exaggerating the gravity of a disease, or in considering an imaginary one as real. The authors of our own time have followed in the traces of preceding nosographists, and especially of Cullen, who has most insisted upon the importance of the disorders of the mind observed in hypochondriasis. It is thus we find Frank, Georget, Falret, Brachet, Dubois, Michea, the authors of the Compendium, place the origin or point of departure of the disease in the brain or nerves. When we peruse these authors, we find the symptom *nosomania*, under the ancient name of hypochondriasis, governing the entire description of the disease; and the different hypochondriacal symptoms, described long ago, as sensation of weight at the heart, intestinal gas, dyspnœa, arterial pulsation, lipothymia, anorexia, &c., are only considered as the sympathetic and more or less immediate effects of the cerebral affection which induces this special mental perversion.

"More than this, the symptoms referrible to the abdomen and thorax, such as eructations, palpitations, &c., which have always been considered to constitute hypochondriasis, and which are even included in the definitions of the nosographists who place the horror or exaggerated fears of disease in such high relief, seem to be considered as no longer necessary to constitute the hypochondriasis of the moderns. It is sufficient for a person to believe himself the subject of a serious disease which he has not, or to be excessively frightened at some slight one that exists, for him to be considered as a hypochondriac. * * * * * * That such an affection exists among the diseases which afflict mankind, cannot be doubted; but it (which we may term *Nosomania*) should be carefully distinguished from the Hypochondriasis of Highmore, Willis, and Hoffmann—although it is yet usually found allied with this latter. In a word, it seems to us we may best understand the relations of *hypochondriasis* and *nosomania* in the way we do those of *canine madness* and *hydrophobia.* It is true that the madness has hydrophobia for a symptom; but we also know that hydrophobia may exist independently of madness, and that even the latter is not necessarily accompanied by it. The individual affected with hypochondriasis is so likewise with nosomania, which is no-wise surprising: for as the symptoms of hypochondriasis indicate affections of almost every system, and to all appearances very grave ones, and as the diseased condition may become very prolonged, the hypochondriac may easily become the victim of exaggerated terrors. But at other times, the nosomania, however well characterized, is no longer connected with hypochondriasis. It may exist as an idiopathic affection, or as a symptom of some very different disease. At other times, all the symptoms of hypochondriasis, as flatulent dyspepsia, arterial murmurs, vertigo, &c. may exist unaccompanied by nosomania. The older physicians made the same distinctions between melancholia and hypochondriasis, which I make between the latter and nosomania They had a hypochondriacal melancholia and melancholia properly so called."

*Nosomania* is in fact a species of Vesania, which may exist in a very slight degree, simply inducing the fear of disease and excessive care of health (*nosophobia*), or become a confirmed mania. In *primary nosomania* the general health may continue good, and no arterial murmurs will be heard; but if this state is prolonged, it may, as any other moral cause, re-act upon the digestive organs, derange their functions, and give rise to confirmed hypochondriasis.

*Symptomatic nosomania* is more common than idiopathic, but it is not exclusively, though usually, observed in hypochondriasis; for it may also exist in scorbutus, cachexia, &c. It may be met with alone or conjoined with melancholia. Its definition may be thus expressed. "*A peculiar form of mental alienation, in consequence of which the individual who is the subject of it is continually and minutely occupied about his health, either believing himself the subject of a disease the presence of which is indicated by no real symptom, or interpreting very slight symptoms into indications of an incurable disease.*" Hypochondriasis may also be thus defined. "*A flatulent dyspepsia necessarily accompanied with arterial murmurs, and frequently with nosomania.*"

### III. General Conclusions.

It results, from all that has gone before, that arterial murmurs are produced by reason of a disproportion existing between the calibre of the vessels and the amount of blood to be propelled through them. The excess of blood arises from a dilatation of the cardiac cavities, induced mechanically, as in the case of insufficiency of the aortic valves, or by atony of the walls of the organ in all the other affections in which arterial murmurs exist. The dilatation is much less in these than in the case of insufficiency, and the existence of præcordial dulness on percussion is therefore only ascertained by comparing its extent during the continuance and after the cessation of the murmurs. (For this comparison, M. Piorry recommends the circumscription of the space over which the dulness extends by means of a black line drawn with ink or nitr. arg.) In this way, we may perceive the coincidence of the existence and intensity of arterial murmurs with the increase of volume of the heart, and *vice versa*. In insufficiency the left ventricle is alone dilated, in diseases dependent upon atony the four cavities are so. In insufficiency, prolonged cachexy, pregnancy and dropsy, the dilatation is accompanied by *marked and evident* hypertrophy; but in affections of a shorter duration, as typhoid fever, variola, &c. it is much less marked, the cavities however still retaining their normal thickness notwithstanding their dilatation. It may be termed *latent* or *diffused*.

" *Hypertrophy*, whether *evident* or *latent*, depending upon atony of the heart, is generally easily cured when the cause of the atony is removed. We can understand this by considering how rapidly the gravid uterus becomes reduced in size after the expulsion of its contents. So in *atonic cardiac affections* the excess of muscular substance disappears, the dilated cavities become narrowed, and at the same time the wave of blood to be propelled is much less considerable. We must not then confound, in regard to its gravity and curability, this form of hypertrophy with that arising from contraction or insufficiency of the cardiac orifices. This last is usually incurable, because the valvular affection which has induced it is beyond the resources of art. In fact, the disease in this case does not consist in the dilatation and hypertrophy of the organ, however considerable they may be, but entirely in the lesion of the orifices. The despairing epigraph affixed to Corvisart's work (*Hæret lateri læthalis arundo*) is thus solely applicable to the affections of the heart connected with disease of its orifices, and does not concern that numerous class derivable from atony of the organ.

" It is necessary to be very chary of bleeding, even when we have to do with a disease of the heart dependent upon lesions of the orifices. Dr. Corrigan has already laid down this precept in respect to cases of insufficiency of the aortic valves, and it should extend to those of narrowing. In both cases, the diminution of the globules by depletion brings on a state of atony, which aggravates the dilatation and hypertrophy dependent upon lesion of the orifices. The same result is observed when the diminution of globules arises spontaneously. The symptoms of disease of the heart, such as dyspnœa, palpitation, &c. are increased in proportion as the organism presents the characters of the cachectic condition. But when this cachexy complicates contraction of the orifices, it is no longer characterized by carotidean murmurs and fulness of pulse; for the wave of blood is now broken in its passage through the narrow orifice, and does not penetrate the arteries with force or volume enough to produce these effects. When, however, the dilatation is excessive and the wave very considerable, it may exert the exaggerated degree of friction against the arterial parietes, notwithstanding this obstruction. At the autopsy of a person who has died of a lesion of the orifice complicated with dropsy, cachexia, &c., we must regard the degree of dilatation and hypertrophy there observed as resulting from a double influence, the lesion of orifice, and the change in the composition of the blood; the first having acted mechanically, the latter by atony."

After noticing the increased volume of the pulse and pulsations of the arteries which coincide with the arterial murmurs, M. Beau offers this cautionary remark.

"The radial pulse is augmented in the same proportion as that of the great arteries. But to appreciate this properly, we must not be content with judging absolutely of the volume of the pulse, which in different individuals varies much according to the calibre of the vessel. Its volume should be compared during the existence of the carotidean murmurs, and after they have ceased. We then find that, however small the radial pulse may have appeared during the continuance of the disease furnishing the murmurs, it becomes still less after their disappearance upon its cure. I may here remark how little care is taken to estimate the volume of the radial pulse. Whenever it is below a certain type with which it is instinctively compared, it is at once concluded that the patient's pulse is small. But this person, whom you see for the first time, may have the radial of a small and even very small calibre; and then the wave dilating it may be as strong and even much stronger than in the normal state, without the pulse losing its conventional character of littleness. The radial artery, like all the others, varies considerably in volume, not only according to the sex, but in persons of the same sex. It is also very often of unequal volume in the two sides of the body. These are facts, the reality of which our anatomical studies prove to us. The carotidean murmurs relieve us from all difficulty in deciding whether the pulse is of normal volume. If the murmurs exist, the volume of the pulse possesses an abnormal plenitude and *vice versâ.*"

The radial pulse, when carotidean murmurs are present, is (1) always of an augmented fulness. 2. It may be (according as the murmurs are intense and the volume of blood propelled considerable) soft, hard, vibrating, or double. 3. The carotidean pulse may be hard, vibrating or double, but it is never soft. 4. When the pulse is hard, vibrating or double at the radial artery, it presents the same characters still more markedly at the carotid. The double pulse here mentioned (*dicrotus* of Galen) is not felt in the radial artery in women, although it is in the carotid. The author attributes it to the elastic re-action of the arch of the aorta in its most forcible degree. The same atony, which produces the dilatation of the cardiac cavities, operates also upon the arterial parietes; so that in the interval of their pulsations, however hard and vibratory these may be, the vessels offer little or no resistance to the finger. As the malady becomes cured however, and the volume of the pulse diminished, the arteries present more tonic resistance to the finger in the interval—the heart propelling less blood into them, and they contracting in a more continuous manner upon that which they contain.

It is owing to this atony of the arteries also that we find them, in the autopsies of individuals dying from diseases in which arterial murmurs had manifested themselves, containing a notable quantity of blood; such congestion arising from their not having possessed at the time of death sufficient tonic power for the expulsion of the blood they contained. Atony likewise in these cases affects various other organs of the economy, as the skin, muscles, &c., but the *iris* is the organ in which it is especially manifested. *The pupil is dilated in all the diseases* (except valvular insufficiency) *in which arterial murmurs exist:* but, as the diameter of this aperture varies much in different persons, to judge of its dilatation we must also examine its condition after the murmurs have disappeared.

But it may be said that, if the excess in the quantity of blood suffices to induce arterial murmurs, these should be found present in *plethora.* Plethora, however, may be *general,* and dependent upon a superabundance of normal blood propelled by a dilated heart, and in such case we shall have the murmurs and other symptoms so often adverted to; but, again, it may be only *local* or *capillary,* congesting the face or various viscera, but not influencing the condition of the general circulation, and therefore not giving rise to any of these phenomena.

Having noticed the diseases which do furnish *arterial murmurs,* the author next briefly glances, by way of contrast, at such as *do not;* and among the foremost of these may be placed the *Phlegmasiæ.* He however confines his observations to *Pneumonia* as the type of other inflammatory affections. He remarks that this is erroneously said to occur only in the plethoric, because the persons whom it attacks may be robust. Moreover, the *pulse* in this disease has been erroneously said to be full or vibratory, which conditions do not exist until the patient has undergone several bleedings and a state of serous polyæmia is consequently induced. The only symptom common to pneumonia, and the diseases characterised by arterial murmurs, is the anxious respiration; but this depends in the one case upon inflammatory hyperæmia of the lung, and in the other upon a non-inflammatory hyperæmia affecting that organ under the influence of the general excess of the blood. In the first or even the second stage of *Phthisis,* we may have the carotidean murmurs, because it is then frequently complicated with a cachexia or hypochondriasis; but when the final stage of the malady arrives, the anormal sounds, with the full pulse and other concomitant symptoms, disappear; as might have indeed been expected from the fact that, at the autopsies of this disease, the heart is found small and contracted.

*Therapeutical Indications.*—Our endeavours should be directed to the remedying that morbid condition of the blood which has induced the atony of tissues, under the influence of which the other symptoms have become developed. When this condition cannot be ascertained with precision, or we have not the means of modifying it, we must content ourselves with watching the progress of the disease and combating its predominant symptoms or any attendant complications. Nevertheless, polyæmia does not demand *blood-letting,* unless a dangerous congestion occur in any of the important viscera; and this may often be preferably treated by Junod's cupping instrument. The only exceptions to this rule are formed by *pregnancy* and simple *amenorrhœa,* in which bleeding produces advantages which are not attended by future ill-effects, that is, if it be not too often repeated. It will be seen then that we *proscribe bleeding just in those affections in which the volume of the pulse is most considerable;* and although this may seem at first sight contradictory, yet it will be found that the best practitioners have always forbidden bleeding, except under special circumstances, in the diseases we have enumerated as characterized by arterial murmurs. Depletion in such cases undoubtedly relieves for the moment, but only to afterwards plunge the patient into a state of adynamia and exhaustion. The development of the pulse and other symptoms in these diseases are due to atony; and, if we bleed, we shall add to the atony causing the disease in question, that form of it which results from the diminution of blood-globules attendant upon loss of blood. The proposition may seem to have a less paradoxical appearance and yet be no less true, if we say that we *must be very careful in detracting blood in those diseases, in which the radial artery does not offer resistance to the finger during the intervals of its pulsations.* Such a condition is always connected with unusual fulness of pulse and arterial murmurs. In *phlegmasiæ,* on the contrary, the pulse is not full at the onset of the disease, and may be even small during its progress, until free bleeding develops and amplifies it. After its anormal elevation under the influence of depletion, it insensibly diminishes in volume with the approach of convalescence, and becomes as small (or rather normal) after the cure as before the attack—the artery recovering, too, its power of resistance to the finger during the intervals of pulsation, which it had lost under the influence of the bleeding.

"The common and precise characters which we have attributed to the diseases in which arterial murmurs are habitally heard, and especially the causes which give rise to these characters, must induce us to look upon all these affections as constituting a separate class in pathology. We then bring to mind certain systems of medicine, which have already more or less signalized such a class under different names. Is it not evident that the *laxum* of Themison, the *alkalescency* of Sylvius, and the *asthenia* of Brown, are terms especially applicable to the diseases which are characterised by arterial murmurs? For these murmurs and their accompanying symptoms depend necessarily upon an *atonic laxity of tissue,* which laxity may often itself result from an *alkaline fluidity* of the blood, and to these are necessarily conjoined an *asthenia,* which, as a general rule, is opposed to the evacuation of blood. And, on the other hand, the *strictum* of Themison, the *acidity* of Sylvius, and the *sthenic* condition of Brown, embrace the diseases which are characterized by an absence of arterial murmurs, especially the phlegmasiæ. These three authors, treating the first on the state of the solids, the second on the composition of the blood, and the third on the consideration of the powers of the economy, had then vaguely prefigured the two general classes of diseases which are physically distinguished by the presence or absence of carotidean murmurs."

Our desire to comprise the account of the whole series of these interesting papers in one article has obliged us to render this entirely analytical, and we must defer to a future opportunity any comments we may have to offer upon the doctrines set forth. In the mean time, however, we believe our readers will agree with us in regarding the Essay a valuable one, and not destitute of important practical bearings.

